# Analysis of the correlation between Zeste enhancer homolog 2 (*EZH2*) mRNA expression and the prognosis of mesothelioma patients and immune infiltration

**DOI:** 10.1038/s41598-022-21005-w

**Published:** 2022-10-04

**Authors:** Kui Fan, Chuan-long Zhang, Bo-hui Zhang, Meng-qi Gao, Yun-chuan Sun

**Affiliations:** 1 Department of Radiation Oncology, Hebei Province Cangzhou Hospital of Integrated Traditional and Western Medicine, No. 31, Huanghe West Road, Yunhe District, Cangzhou, 061000 Hebei China; 2 Guang’anmen Hospital, China Academy of Chinese Medical Sciences, Beijing, 100053 China; 3 Department of Neurology, Cangzhou Hospital of Integrated Traditional Chinese and Western Medicine-Hebei Province, Cangzhou, 061000 Hebei China; 4 Wangjing Hospital, China Academy of Chinese Medical Sciences, Beijing, 100102 China

**Keywords:** Cancer, Computational biology and bioinformatics

## Abstract

Mesothelioma lies one of the most malignant tumors, in which the identification of the corresponding biomarkers is extremely critical. This study aims to investigate the prognostic value of enhancer homolog 2 (*EZH2*) mRNA expression in mesothelioma patients accompanied with its immune infiltration analysis. Gene expression, clinical information and enrichment analysis were obtained based on the Cancer Genome Atlas (TCGA), the immune infiltration analysis and bioinformatics analysis were performed. Clinical information and gene expression were obtained from 86 patients with mesothelioma based on TCGA database. Survival analysis, GSEA enrichment analysis, and immune infiltration analysis of *EZH2* expression were carried out using R (version 3.6.3) (statistical analysis and visualization). The correlation of *EZH2* expression with immune cell infiltration in mesothelioma was analyzed according to the TIMER database (Fig. https://cistrome.shinyapps.io/timer/). A univariate and multivariate analysis of general data obtained from the TCGA database was performed, involving age, gender, stage, pathological type, and whether they had received radiotherapy, the results indicated the association of high expression of *EZH2* with poor prognosis in mesothelioma patients, with the worse prognosis in the High group (HR = 2.75, 95% CI 1.68–4.52, *P* < 0.010). Moreover, ROC curves showed that *EZH2* expression predicted 1-year survival with an AUC of 0.740, 2-year survival with an AUC of 0.756, and 3-year survival with an AUC of 0.692, suggesting a robust predictive effect of *EZH2* expression on prognosis. KEGG pathway analysis indicated five pathways showing the strongest positive correlation with *EZH2* expression: cell cycle, DNA replication, Cell adhesion molecules cams, Primary immuno deficiency, Tsate transduction, and five pathways showing the strongest negative correlation with *EZH2* expression: Glycolysis gluconeogenesis, Drug metabolism, cytochrome P450, retinol metabolism, fatty acid metabolism ribosome. We investigated the correlation between *EZH2* expression and the level of immune infiltration in mesothelioma tissues. The results indicated that *EZH2* expression played a critical role in immune infiltration, of which the high expression was correlated with the reduced number of NK cells, Mast cells, and Th17 cells. Moreover, mesothelioma patients with high *EZH2* expression differ from those with low *EZH2* expression in their tumor immune microenvironment. *EZH2*, as a new prognostic biomarker for mesothelioma, contributes to elucidating how changes in the immune environment promote the development of mesothelioma. Further analysis, *EZH2* may serve as a biological test to predict the prognosis of mesothelioma.

## Introduction

Mesothelioma is described as a malignant tumor arising from the mesothelial surface of the pleural cavity, peritoneal cavity, testicular tunica vaginalis, or pericardium, of which 80% of cases originate from the pleura^1^. The disease as a rare malignancy possess a poor prognosis and overall survival (OS) of approximately 9–17 months after diagnosis^1^, particularly in patients with pleural mesothelioma where the effect is not obvious due to its low incidence in mesothelioma despite some related studies, chemotherapy, immunization, and local radiotherapy. The identification of molecular biomarkers predictive of prognosis is limited by the small number of mesothelioma patients who have been involved in clinical trials as well as the heterogeneity of the study population. Therefore, it is significant to identify the therapeutic targets for this malignancy.

*EZH2* and Embryonic Ectoderm Development (*EED*) encoding polycomb repression complex-2 (PRC-2) have been demonstrated to be overexpressed in MPM lines, occupying about 85% of Mesothelioma in comparison with normal pleura, which is related to the reduced survival rate of patients^2^. *EZH2* plays roles in manipulating gene expression to inhibit the differentiation of stem cells and progenitor cells, of which the abnormal activity is considered to be the driving factor of carcinogenesis. The studies of *EZH2* mRNA are in full swing recently, with its mechanism demonstrated to be associated with silencing epigenetic genes^3^. Studies have indicated that the abnormal expression of *EZH2* is associated with poor prognosis in various malignancies such as lung^4^. *EZH2* manipulates gene expression to prevent the differentiation of stem and progenitor cells, of which the abnormalities in activity are considered to be drivers of carcinogenesis. While the monotherapy as an *EZH2* inhibitor showed robust antitumor activity in the treatment of anti-mesothelioma, with good safety/tolerability^5^. Macrophages can directly deliver cytotoxicity to mesothelioma cells, without phagocytosis^6^. Tumor-associated macrophages (TAM) serve as the drivers of Mesothelioma growth, progression, and drug resistance to Tazemetostat. The therapeutic effect of *EZH2* pharmacological inhibition can be enhanced by evaluating the TAM consumption strategies^7^. The robust safety and tolerance of tazemetostat were initially demonstrated by a multicenter, open-label, phase 2 study of *EZH2* inhibitor tazemetostat in patients with recurrent or refractory BAP1-inactivated malignant pleural mesothelioma. In comparison to traditional second-line cytotoxic drugs, involving gemcitabine, vinorelbine and even immunotherapy: nivolumab plus ipiliminumab can achieve the elevated response rate and progression-free survival (PFS)^8^. The inactivation of BAP1 induces the upregulation of BAP1 and the dependence on methyltransferase *EZH2*, which trimethylates lysine 27 on histone H3 (H3K27me3) as one of the chromatin modified PRC-2^9,10^. The lack of BAP1 will induce mesothelioma cells sensitive to *EZH2* drug inhibition^9^.

However, the studies on the correlation of *EZH2* with mesothelioma are still lacked, remaining the role in mesothelioma unknown. In this paper, the expression of *EZH2* was statistically analyzed for the prognosis of mesothelioma depending on the clinical data from the TCGA database, and the correlation between *EZH2* and immune infiltration in patients with mesothelioma was statistically analyzed, so as to further elaborate the potential mechanism of *EZH2*’s immune impact in this disease (tumor microenvironment) and seek the relevant impact mechanism. Our work revealed that the increased expression of *EZH2* was linked to poor OS among Mesothelioma patients. GSEA pathways indicated that Cell cycle, DNA replication, Primary immuno deficiency, Metabolism were all involved in the association with *EZH2* expression. Moreover, a connection was identified between *EZH2* and tumor-infiltrating immune cells. The presented work here provides a detailed analysis of the role of *EZH2* in Mesothelioma development and indicates mesothelioma prognosis, which contributes to the understanding of the underlying mechanisms of Mesothelioma.

## Materials and methods

### Evidence from TCGA database

The analyses are based on TCGA (https://portal.gdc.cancer.gov), which was obtained from the MESO (mesothelioma) project with immune system infiltration, gene expression (HTSeq-FPKM), and related clinical information^11^. Samples missing or lacking sufficient data in terms of age, TNM stage, OS time, and distant and lymph node metastasis were excluded from the study. RNA-Seq and clinical data were retained for further study. Our study conformed to the publication guidelines provided by TCGA.

### Survival analysis

Based on the clinical information obtained from the TCGA database, the correlation of the *EZH2* expression (high and low expression in median) with mesothelioma patient survival was analyzed using the software: R (version 3.6.3) (statistical analysis and visualization) R package: survminer package [0.4.9 version] (for visualization), survival package [3.2-10 version] (for statistical analysis of survival data); survival curves were plotted. Statistical criteria were: P < 0.05.

### GSEA enrichment analysis

We performed GSEA on RNA-Seq data obtained from TCGA^12^. The number of permutations was set to 1,000. KEGG pathways were analyzed to investigate the possible biological functions of *EZH2*^13–15^. The statistical criteria were: false discovery rate (FDR) < 0.05 and *P*-value < 0.05.

### Immune infiltrate analysis

Related modules were utilized to analyze the potential relationship between *EZH2* expression and mesothelioma immune infiltration by TIMER (Fig. https://cistrome.shinyapps.io/timer/). We examined the *EZH2* expression in mesothelioma tissues and its relationship with the abundance of immune infiltrates by gene modules, involving CD4 T+ cells, dendritic cells, B cells, CD8+ T cells, neutrophils, and macrophages. The relationship between gene expression levels and immune infiltration was also plotted.

### Statistical analysis

Statistical analysis was performed using R (version 3.6.3) (Statistical Analysis and Visualization). For each level in the measurement data meeting, the chi-square test was adopted in the conditions that the theoretical frequency is greater than 5 and the total sample size is greater than 40. Otherwise, Fisher’s exact test shall be used. In the enumeration data meeting the normal distribution, the chi-square test was selected, otherwise the Wilcoxon rank sum test was selected. Log-rank test, Cox regression analysis, and Survival curve were performed for high and low *EZH2* mRNA expression according to the survival time in the database. The results at *P* < 0.05 were considered statistically significant.

### Ethics approval and consent to participate

The data for this study come from the TCGA database and do not require ethical approval.

## Results

### Correlation between EZH2 expressions and clinicopathologic features of mesothelioma

Based on, we divided the 86 patients downloaded from the TCGA database into high expression group and low expression group according to the median *EZH2*, covering 43 patients with high expression and 43 patients with low expression, with Patients’ pathologic clinico-characteristics listed in Table 1.

**Table 1:** Summary of the clinicopathological characteristics of mesothelioma patients for *EZH2* expression.

Characteristic	Low expression of *EZH2*	High expression of *EZH2*	*P*
n	43	43	
**Gender, n (%)**			1.000
Female	7 (8.1%)	8 (9.3%)	
Male	36 (41.9%)	35 (40.7%)	
**Age, n (%)**			0.665
≤ 65	25 (29.1%)	22 (25.6%)	
> 65	18 (20.9%)	21 (24.4%)	
**Histological type, n (%)**			0.381
Biphasic	9 (10.5%)	13 (15.1%)	
Diffuse malignant	3 (3.5%)	2 (2.3%)	
Epithelioid	31 (36%)	26 (30.2%)	
Sarcomatoid	0 (0%)	2 (2.3%)	
**Residual tumor, n (%)**			0.123
R0	11 (32.4%)	5 (14.7%)	
R1	2 (5.9%)	1 (2.9%)	
R2	5 (14.7%)	10 (29.4%)	
**History asbestos exposure, n (%)**			1.000
No	7 (10%)	7 (10%)	
Yes	27 (38.6%)	29 (41.4%)	
**Pathologic stage, n (%)**			0.214
Stage I	2 (2.3%)	8 (9.3%)	
Stage II	10 (11.6%)	6 (7%)	
Stage III	23 (26.7%)	21 (24.4%)	
Stage IV	8 (9.3%)	8 (9.3%)	
**T stage, n (%)**			0.081
T1	4 (4.8%)	10 (11.9%)	
T2	18 (21.4%)	8 (9.5%)	
T3	14 (16.7%)	17 (20.2%)	
T4	7 (8.3%)	6 (7.1%)	
**N stage, n (%)**			0.067
N0	18 (22%)	25 (30.5%)	
N1	6 (7.3%)	4 (4.9%)	
N2	17 (20.7%)	9 (11%)	
N3	0 (0%)	3 (3.7%)	
**M stage, n (%)**			0.599
M0	26 (44.1%)	30 (50.8%)	
M1	2 (3.4%)	1 (1.7%)	
**Radiation therapy, n (%)**			0.685
No	31 (36.5%)	29 (34.1%)	
Yes	11 (12.9%)	14 (16.5%)	
**Laterality, n (%)**			1.000
Left	16 (19.3%)	14 (16.9%)	
Right	27 (32.5%)	26 (31.3%)	
**OS event, n (%)**			0.016
Alive	11 (12.8%)	2 (2.3%)	
Dead	32 (37.2%)	41 (47.7%)	
**DSS event, n (%)**			0.005
Alive	17 (26.2%)	5 (7.7%)	
Dead	16 (24.6%)	27 (41.5%)	
**PFI event, n (%)**			0.154
Alive	16 (18.6%)	9 (10.5%)	
Dead	27 (31.4%)	34 (39.5%)	
**Age, meidan (IQR)**	62 (55, 69.5)	65 (60, 68)	0.536

*OS* overall survival, *DSS* disease free survival, *PFI* progression-free interval.

### Survival outcomes and analysis variables

**Figure 1:**
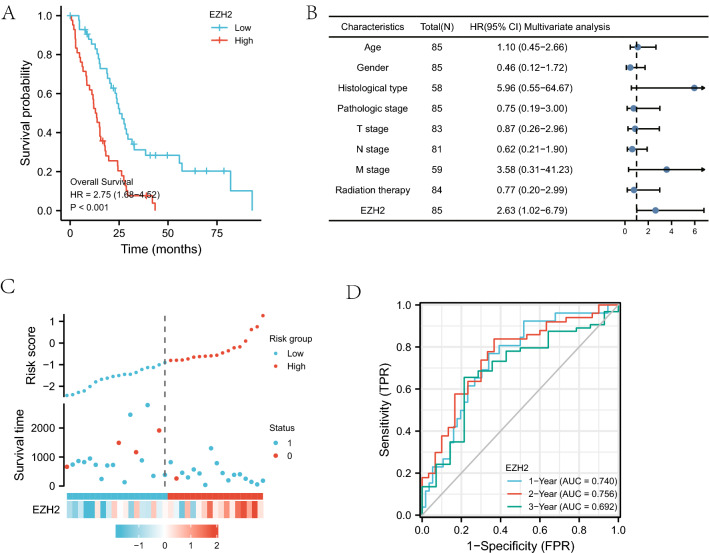
(**A**) Kaplan–Meier survival curves for mesothelioma patients, stratified by *EZH2* expression levels. (**B**) Multivariate Cox analysis of expression and other clinicopathological variables. (**C**) *EZH2* expression distribution and survival status. (**D**) ROC curves of *EZH2*.

The results of the survival analysis of *EZH2* expression group indicated that the survival time was 26.3 (23–32.7) months in the low expression group, and 13.7 (9.9–17.9) months in the high expression group. Statistical tests were performed on the data of the two groups: the results of the log-rank test suggested the statistically significant difference in the survival time distribution of group groups (*P* < 0.001), which was consistently indicated by the results of Cox regression (*P* < 0.001). The high expression group had a worse prognosis (HR = 2.75, 95% CI 1.68–4.52, *P* < 0.001)(Fig. 1A).

Cox analysis was chosen to explore the relationship between *EZH2* expression and OS as well as other multivariate characteristics in mesothelioma patients. Both univariate and multivariate analyses indicated the association of high expression of *EZH2* with poor prognosis in mesothelioma patients (Table 2). Univariate correlation analysis showed significant association of *EZH2* expression with OS (*P* < 0.001), and the pathological type (HR = 5.96, 95% CI 0.55–64.67, *P* = 0.020) and *EZH2* expression (HR = 2.35, 95% CI 1.62–3.42, *P* < 0.001) were significantly associated with OS in mesothelioma patients. The multivariate Cox regression forest plot was drawn according to the results of multivariate analysis, from which it could be concluded that *EZH2* expression acted as an independent prognostic factor for the survival of mesothelioma patients (HR = 2.63, 95% CI 1.02–6.79, *P* = 0.046) (Fig. 1B). The distribution of *EZH2* expression, survival of mesothelioma patients, and *EZH2* expression are depicted in Fig. 1C. The expression of *EZH2* provided a robust predictive effect on prognosis as ROC curves showed the AUC of 0.740 in *EZH2* expression predicting 1-year survival, 0.756 in 2-year survival, and 0.692 in 3-year survival (Fig. 1D).

**Table 2:** Correlation between OS and multivariable characteristics in TCGA patients via Cox regression: Univariate survival model and Multivariate survival model.

Characteristics	Total (N)	Univariate analysis		Multivariate analysis	
		Hazard ratio (95% CI)	*P* value	Hazard ratio (95% CI)	*P* value
**Age**	85				
≤ 65	46	1.27(0.81–2.09)	0.286	1.10 (0.45–2.66)	0.834
> 65	39				
**Gender**	85				
Female	15	0.94 (0.52–1.73)	0.850	0.46 (0.12–1.72)	0.247
Male	70				
**Histological type**	58				
Epithelioid	57	13.49 (1.51–120.73)	0.020	5.96 (0.55–64.67)	0.142
Sarcomatoid	1				
**Pathologic stage**	85				
Stage I & Stage II	26	0.97 (0.58–1.65)	0.923	0.75 (0.19–3.00)	0.678
Stage III & Stage IV	59				
**T stage**	83				
T1&T2	39	0.96 (0.59–1.55)	0.852	0.87 (0.26–2.96)	0.823
T3&T4	44				
**N stage**	81				
N0&N1	53	0.90 (0.54–1.51)	0.690	0.62 (0.21–1.90)	0.408
N2&N3	28				
**M stage**	59				
M0	56	1.92 (0.45–8.09)	0.376	3.58 (0.31–41.23)	0.306
M1	3				
**Radiation therapy**	84				
No	59	0.70 (0.41–1.18)	0.174	0.77 (0.20–3.00)	0.707
Yes	25				
BAP1	85	1.05 (0.84–1.31)	0.67	0.94 (0.60–1.47)	0.786
*EZH2*	85	2.35 (1.62–3.42)	< 0.001	2.64 (1.02–6.79)	0.046

### GSEA enrichment analysis

**Figure 2:**
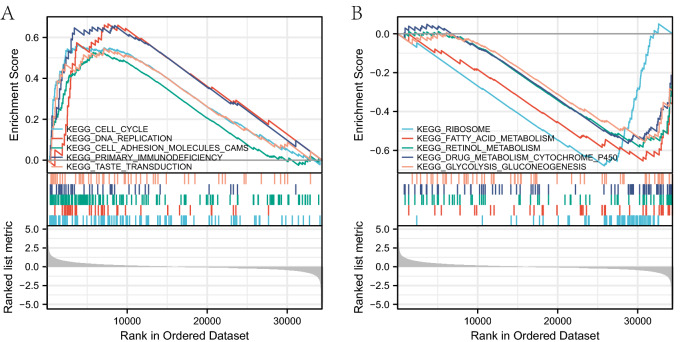
KEGG pathway showed five positively correlated groups and five negatively correlated groups.

The GSEA software was utilized to perform KEGG pathway analysis to investigate the potential biological functions of *EZH2*. The most highly enriched signaling pathways were selected according to the normalized enrichment fraction (NES). As shown in Table 3, five pathways exhibited the strongest positive correlation with *EZH2* expression: cell cycle pathway, DNA replication pathway, cell adhesion molecule pathway, primary immunodeficiency pathway, and taste conduction pathway; and five with the strongest negative correlation with *EZH2* expression: glycolytic gluconeogenesis pathway, drug metabolism cytochrome P450 pathway, retinol metabolism pathway, fatty acid metabolism pathway, and ribosome pathway (Fig. 2). These results suggested the essential significance of pathways regulating cell cycle control and fatty acid metabolism, glycolysis, and gluconeogenesis in mesothelioma patients, which are closely associated with *EZH2* expression.

**Table 3:** Signaling pathways most significantly correlated with *EZH2* expression based on their normalized enrichment score (NES) and p-value, normalized enrichment score (NES).

	ID	NES	P value	p.adjust
Positive	KEGG_CELL_CYCLE	2.091182	0.001626	0.043625
	KEGG_DNA_REPLICATION	1.987745	0.001799	0.043625
	KEGG_CELL_ADHESION_MOLECULES_CAMS	1.951839	0.001645	0.043625
	KEGG_PRIMARY_IMMUNODEFICIENCY	1.947583	0.001821	0.043625
	KEGG_TASTE_TRANSDUCTION	1.733792	0.003552	0.052474
Negative	KEGG_GLYCOLYSIS_GLUCONEOGENESIS	− 1.915402	0.002304	0.043625
	KEGG_DRUG_METABOLISM_CYTOCHROME_P450	− 1.958930	0.002342	0.043625
	KEGG_RETINOL_METABOLISM	− 2.004915	0.002304	0.043625
	KEGG_FATTY_ACID_METABOLISM	− 2.094151	0.002237	0.043625
	KEGG_RIBOSOME	− 2.455635	0.002475	0.043625

*NES* normalized enrichment score.

### Immune infiltration

**Figure 3:**
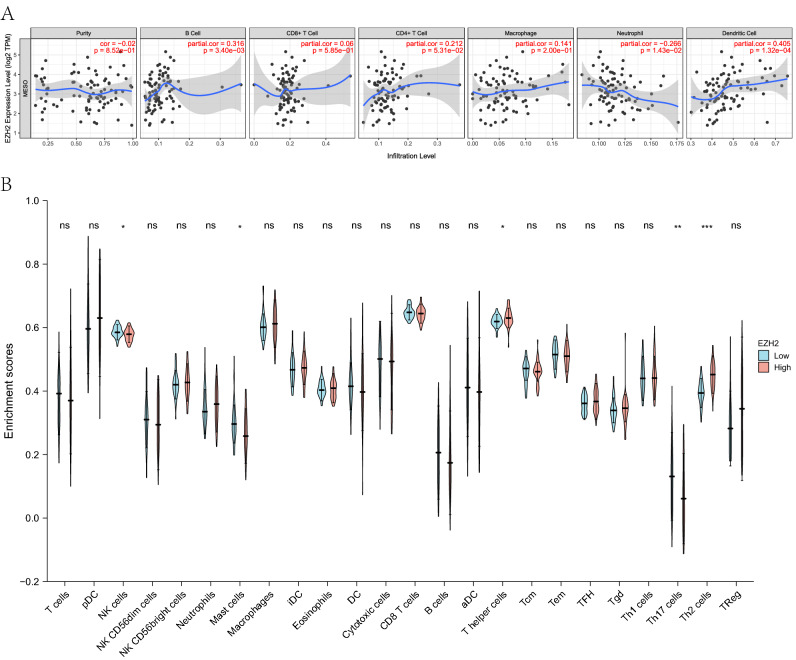
(**A**) Correlations between *EZH2* expression and immune infiltration levels. (**B**) The varied proportions of 24 subtypes of immune cells in high and low *EZH2* expression groups in tumor samples.

Independent tumor-infiltrating lymphocytes play crucial roles in the prediction of OS and lymph node metastasis. Therefore, we analyzed the correlation of *EZH2* expression with the level of immune infiltration in mesothelioma tissues using TIMER. As depicted in Fig. 3A, the positive correlation was indicated of EZH2 expression with the levels of B cells (p-value = 3.40 × 10^−3^), CD4 + T cells (p-value = 5.31 × 10^−2^), and Dendritic cells (p-value = 1.32 × 10^−4^), indicating a key role of *EZH2* expression in immune infiltration. Furthermore, we also sought to determine whether there lies a difference in the tumor immune microenvironment between mesothelioma patients with high or low *EZH2* levels. The 86 tumor specimens were divided into two groups, 43 in the high expression group and 43 in the low expression group. The R package was adopted: GSVA package [version 1.34.0]^16^ to download the gene expression profiles of the samples to determine the levels of 24 immune cells, in which the GSEA algorithm applied to 24 immune cell subtypes contributed to assessing the differences in expression levels in the two groups. In Natural Killer (NK) cells, High was lower than Low, with statistically significant difference (*P* = 0.023); in Mast cells, High was lower than Low, with statistically significant difference (*P* = 0.040); in T helper (TH) cells, High was higher than Low, with statistically significant difference (*P* = 0.021); in Th17 cells, High was lower than Low, with statistically significant difference (*P* = 0.005); in Th2 cells, High was higher than Low, with statistically significant difference (*P* < 0.001) (Fig. 3).

## Discussion

Malignant mesothelioma is a rare primary cancer with survival of less than 1 year^1^. A trimodality treatment approach in combination of surgery, chemotherapy, and sequential radiation therapy (RT) represents the mainstream of current mesothelioma treatment options^17^. While the survival still remains unimproved, urgently requiring the prognostic biomarkers to guide clinical decision-making. The TCGA database can provide a large amount of data from mesothelioma patients, through which new potential prognostic biomarkers can be searched for.

*EZH2* as the catalytic subunit of polycomb repressive complex 2 (PRC2) represses target genes by H3K27me3^18^, of which the highly activated mutations frequently locate in the early stages of cancer, suggesting a role for activated PRC2 cancer drivers. Excessive *EZH2* expression alters histone modifications, leading to an aberrant epigenetic landscape that may contribute to cancer progression^19^. Based on this, we carried out the correlation between *EZH2* mRNA levels and the prognosis of mesothelioma patients and immune infiltration analysis depending on the TCGA database.

We performed a univariate and multivariate analysis of the collated general data obtained from the TCGA database, covering age, gender, stage, pathological type, and whether they had received radiotherapy, demonstrating the association of high expression of *EZH2* with poor prognosis in mesothelioma patients. The results of the survival analysis showed that the survival time was 26.3 (23–32.7) months in low expression group, while 13.7 (9.9–17.9) months in high expression group was, indicating that the *EZH2* expression acted as an independent prognostic factor for the survival of mesothelioma patients (HR = 2.63, 95% CI 1.02–6.79, P = 0.046) (Fig. 1B). As the ROC curves showed in Fig. 1A, *EZH2* expression predicted 1-year survival with an AUC of 0.740, 2-year survival with an AUC of 0.756, and 3-year survival with an AUC of 0.692 (Fig. 1D), indicating a good predictive effect of *EZH2* expression on prognosis. To further investigate the mechanism of *EZH2* action, we performed the KEGG pathway analysis by GSEA software to explore the potential biological function of *EZH2*. KEGG pathway analysis reported five pathways lying the strongest positive correlation with *EZH2* expression: cell cycle, DNA replication, Cell adhesion molecule, primary immunodeficiency, and taste conduction; and five pathways lying the strongest negative correlation with *EZH2* expression: glycolytic gluconeogenesis pathway, drug metabolism cytochrome P450 pathway, retinol metabolism pathway, fatty acid metabolism pathway, and ribosome pathway (Fig. 2). These results suggest the essential roles of pathways regulating cell cycle control and fatty acid metabolism, glycolysis, and gluconeogenesis in mesothelioma patients, which are closely associated with *EZH2* expression.

The complex interplay between tumors and their microenvironment remains to be elucidated. Recently, the immune infiltrating components have been found to vary at every tumor stage, where the specific cells significantly impact on survival. The tumors progress is accompanied by the increased density of TH cells and NK cells, while the decreased density of most T cells. B cells serve as a key role in the core immune network and are associated with prolonged survival, while play a dual role in promoting tumor recurrence and progression with high expression in advanced patients, thus exhibiting a dual role in tumor development. Our results indicated that *EZH2* was closely related to the survival of mesothelioma patients. In order to explore the relationship between *EZH2* and tumor immune microenvironment, we analyzed the correlation of *EZH2* expression in mesothelioma tissues with immune infiltration level using TIMER, demonstrating the crucial role of *EZH2* expression in immune infiltration. In addition, we also determined whether existing a difference in the tumor immune microenvironment between mesothelioma patients with high or low *EZH2* levels. Among them, in NK cells, the High expression group was lower than the low expression group (*P* = 0.023). In Mast cells, the high expression group was lower than the low expression group (*P* = 0.040). In TH cells, the high expression group was higher than the low expression group (*P* = 0.021). In Th17 cells, the high expression group was lower than the low expression group (P = 0.005). In Th2 cells, the high expression group was higher than the low expression group (P < 0.001).

There appears increasing evidence that *EZH2* can not only inhibit tumor genes but also play roles in manipulating collective immune homeostasis as well as immune-related cells, especially in the development, differentiation, and function of T cells^20^. *EZH2* has the certain regulatory effects on T cell differentiation and epigenetic inheritance of Treg function. The pharmacological inhibition of *EZH2* in human T cells using CPI-1205 resulted in phenotypic and functional alterations in Tregs, enhancing the cytotoxic activity of Teffs. The regulation of *EZH2* expression in T cells could alleviate the antitumor response elicited by anti-CTLA-4 treatment^21^. *EZH2* inhibition was demonstrated to restore the cytotoxic response of CD8 T cells in patients with systemic lupus erythematosus (SLE), inhibiting the incidence of infection and thus reducing death resulting from infection^22^. Shane et al. showed that the phosphorylation status of *EZH2* determines its property to maintain anti-tumor immunity in CD8+ T memory precursor cells, and Akt-mediated *EZH2* phosphorylation lies a key target for enhancing anti-tumor immunotherapy strategies^23^. Combined with the recent data published in Mesothelioma Immunotherapy deserves our attention (https://www.mesothelioma.com/treatment/immunotherapy/), we learned that immunotherapy helps to improve the survival time of mesothelioma patients and maximize the efficacy of first-line treatments, such as chemotherapy and radiotherapy, but how to seeking optimal options in mesothelioma treatment remains a focus of future research, and modulation of the immune microenvironment may be one of these options.

In addition, *EZH2* has also been demonstrated associated with tumor resistance and its inhibitors can overcome resistance to immunotherapy^24,25^. *EZH2* as a major driving force in cancer cell immunoediting mediates immune escape by down-regulating immune recognition and activation, up-regulating immune checkpoints, thus generating an immunosuppressive tumor microenvironment^26^. The knockdown or inhibition of *EZH2* upregulated the MET expression and phosphorylation, modifying cell proliferation and EGFR-TKI resistance in vitro. The inhibition of MET or PI3K/AKT elevated *EZH2* levels and restored sensitivity to EGFR-TKIs. These findings suggest that the “MET-AKT-EZH2” feedback loop manipulates EGFR-TKI resistance^27^. *EZH2* was also highly expressed in the lung cancer with positive KRAS expression, exhibiting a positive correlation, as well as with the expression of BRAF, especially in lung squamous cell carcinoma. High expression of *EZH2* possibly possess a synergy with KRAS and BRAF mutations^4^.

There exists a correlation between *EZH2* and the level of H3K27me3 modification, in which *EZH2* as a subunit of PRC2 trimethylates H3K27 in the nucleus. PRC1 binds to PRC2-modified H3K27me3 and ubiquitinated histone H2A at lysine 119, facilitating the chromatin compaction and transcriptional silencing of downstream genes, as well as playing roles in the tumor^28^. Malignant mesothelioma as a highly invasive cancer, is generally diagnosed at an advanced stage. Therefore, highly sensitive and specific markers are highly required for early diagnosis. The deletion of BAP1 and high expression of *EZH2* were both observed in malignant mesothelioma, which were merely observed in benign lesions. The combination of the two signals can serve as a high sensitive and specific a marker contributing to differentiating epithelioid/biphasic malignant mesothelioma from benign mesothelial lesions. Survival analysis indicated the missed *BAP1*, while not high expression of *EZH2*, which indicated a better prognosis. This combination elevated the diagnostic accuracy. Epigenetic regulation is adopted as a new method for cancer treatment, among which the inhibition of *EZH2* can provide targeted epigenetic regulation. The relation of the abnormal expression of *EZH2* with poor prognosis has been demonstrated in a variety of malignant tumors. *EZH2* regulates gene expression to prevent the differentiation of stem cells and progenitor cells, of which the abnormal activity is considered to act as the driving factor of carcinogenesis. Currently, the research on epigenetics involving *EZH2*, DNA methyl transferases (DNMTs), as well as histone methyl transferases (HMTs) has absorbed increasing attention, with their corresponding inhibitors displaying great value in cancer treatment. Studies have indicated that the dual inhibition of DNMTs and *EZH2* can eliminate the inherent and acquire drug resistance of myeloma cells to IMiDs in a brain-independent way^29^, which will perhaps play a more contributing role in the treatment of malignant mesothelioma, which is worthy of further study and exploration^30^.

*EZH2* can manipulate the metabolic activity of tumor cells through epigenetic regulation, which in turn affects tumor progression^31^. The famous Warburg Effect indicates that the glucose metabolism can be remodeled by cancer cells even under hypoxia^32^. According to our results, the expression of *EZH2* is closely linked to the glycolytic gluconeogenesis pathway. Brookes et al. found that the up-regulation of *EZH2* can elevate the intracellular deoxyglucose levels and induce a slight increase in mitochondrial oxidative capacity. However, cells overexpressing *EZH2* may also obtain energy mainly rely on enhanced glycolysis as source. *EZH2* can promote tumorigenesis and malignant progression by activating glycolysis through the EAF2-HIF1α signaling axis^33^. Accumulating evidence demonstrates that *EZH2* as the catalytic subunit of PRC2 also directly or indirectly plays a key role in the metabolic process of cancer cells^31^. Recent studies have indicated the significantly downregulated EZH2 expression in response to glucose deprivation in glucose-sensitive colorectal cancer cell lines in which the *EZH2* knockout cells are more resistant to glucose deprivation. *EZH2* deficiency upregulates glutaminase (GLS) expression and promotes glutamate production, leading to the increased intracellular glutathione (GSH) synthesis and glucose deprivation-induced reactive oxygen species (ROS)-mediated cell death is ultimately attenuated^34^.

## Conclusion

High expression of *EZH2* predicts a poor prognosis in mesothelioma or can serve as a prognostic indicator and target gene in mesothelioma. The mechanism may be linked to immune infiltration and metabolism. The increasing attention to the expression of EZH2 and the immune microenvironment in mesothelioma may significantly contribute to enhancing prognosis, which is worthy of further exploration.

## Data Availability

The datasets used and/or analyzed during the current study are available from the TCGA. Our study conformed to the publication guidelines provided by TCGA.

## Abbreviations

EZH2: The prognostic value of enhancer homolog 2
TCGA: The Cancer Genome Atlas
OS: Overall survival
DSS: Disease free survival
PFI: Progression-free interval
NES: Normalized Enrichment Score
PRC2: Polycomb repressive complex 2
